# Primary Pulmonary Choriocarcinoma Treated With Neoadjuvant Chemotherapy and Lobectomy: A Case Report

**DOI:** 10.7759/cureus.21931

**Published:** 2022-02-05

**Authors:** Mohammad M Dlewati, Teresita Gonzalez, Syed S Razi, Syeda F Hussain, Jacqueline Bennett

**Affiliations:** 1 Internal Medicine, Memorial Healthcare System, Hollywood, USA; 2 Thoracic Surgery, Memorial Healthcare System, Hollywood, USA; 3 Pathology, Memorial Healthcare System, Hollywood, USA

**Keywords:** beta-hcg, primary pulmonary choriocarcinoma, vip chemotherapy, novel chemotherapy regimen, pulmonary choriocarcinoma, extragonadal germ cell tumors

## Abstract

Primary pulmonary choriocarcinomas (PPC) are a rare form of extragonadal germ cell tumors (GCT). They present as lung nodules and secrete beta-human chorionic gonadotropin (β-HCG). This is a rare case of PPC that presented insidiously in a postmenopausal woman. Clinical suspicion arose due to markedly elevated serum β-HCG and lung tumor biopsy immunohistochemical staining negative for markers of small cell and non-small cell carcinomas of the lung. The diagnosis of PPC was made after staining positive for markers of GCTs including β-HCG in the absence of a primary tumor in the reproductive organs. The patient was treated with neoadjuvant vincristine, ifosfamide, and cisplatin (VIP) chemotherapy, followed by video-assisted thoracoscopic surgery (VATS) with lobectomy and mediastinal lymph node dissection. This is the first reported case of PPC treated with VIP induction chemotherapy. The patient initially had complete pathologic response and remission; however, she presented with relapse at a nine-month follow-up with new pulmonary nodules and metastatic disease to the brain.

## Introduction

Extragonadal germ cell tumors (GCTs) are a heterogeneous group of tumors arising from extragonadal locations without evidence of a gonadal primary. Choriocarcinoma is nonseminomatous and is among the rarest of GCTs. It comprises syncytiotrophoblast cells that secrete β-human chorionic gonadotropin (β-HCG). Although choriocarcinoma is known to metastasize early to the lungs, primary pulmonary choriocarcinoma (PPC) is extremely rare. Only ~65 cases have been reported in the literature, including many diagnosed after autopsy [1-3]. This is a case of a malignant GCT, non-dysgerminoma with choriocarcinoma component, meeting criteria for PPC. In the absence of a standardized protocol or formal guidelines for the proper treatment of PPC, remission has been achieved by a combination of surgery and chemotherapy regimens adopted from the treatment of high-risk GCTs.

## Case presentation

**Figure 1:**
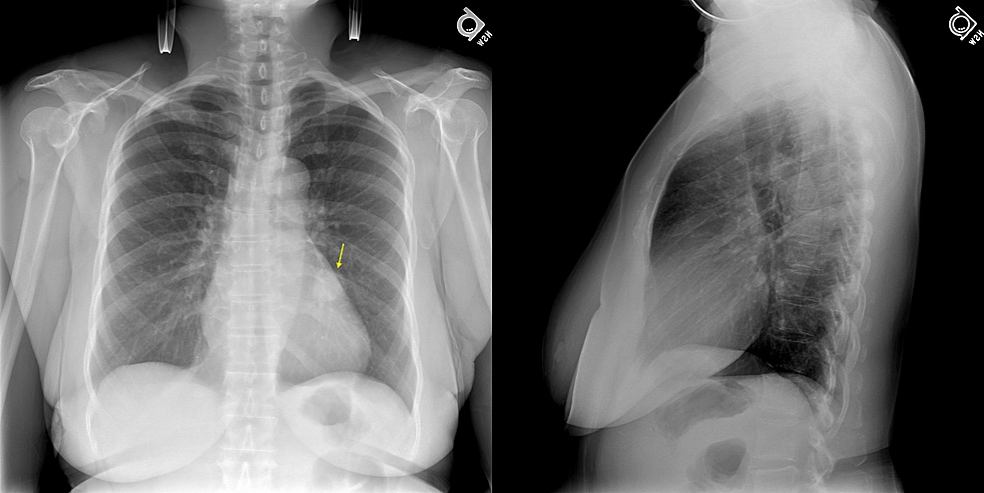
Posteroanterior (PA) and lateral X-ray revealed a 2.5-cm rounded lesion in the left infra-hilar region best seen on the PA film.

A 54-year-old postmenopausal female presented to the ED with dyspnea, cough, and intermittent hemoptysis. Physical exam was notable for normal oxygen saturation of 98%, a regular respiratory rate of 16 with unlabored breaths, and clear breath sounds to auscultation. A posteroanterior (PA) and lateral X-ray revealed a 2.5-cm rounded lesion in the left infra-hilar region (Figure 1). Her serum β-HCG had been increasing for two years and now elevated to 5,057 mIU/mL. An evaluation by a gynecologic oncologist had ruled out gynecologic malignancy. Her routine lab work was otherwise unremarkable. Her medical history was significant for hypertension and ischemic stroke.

**Figure 2:**
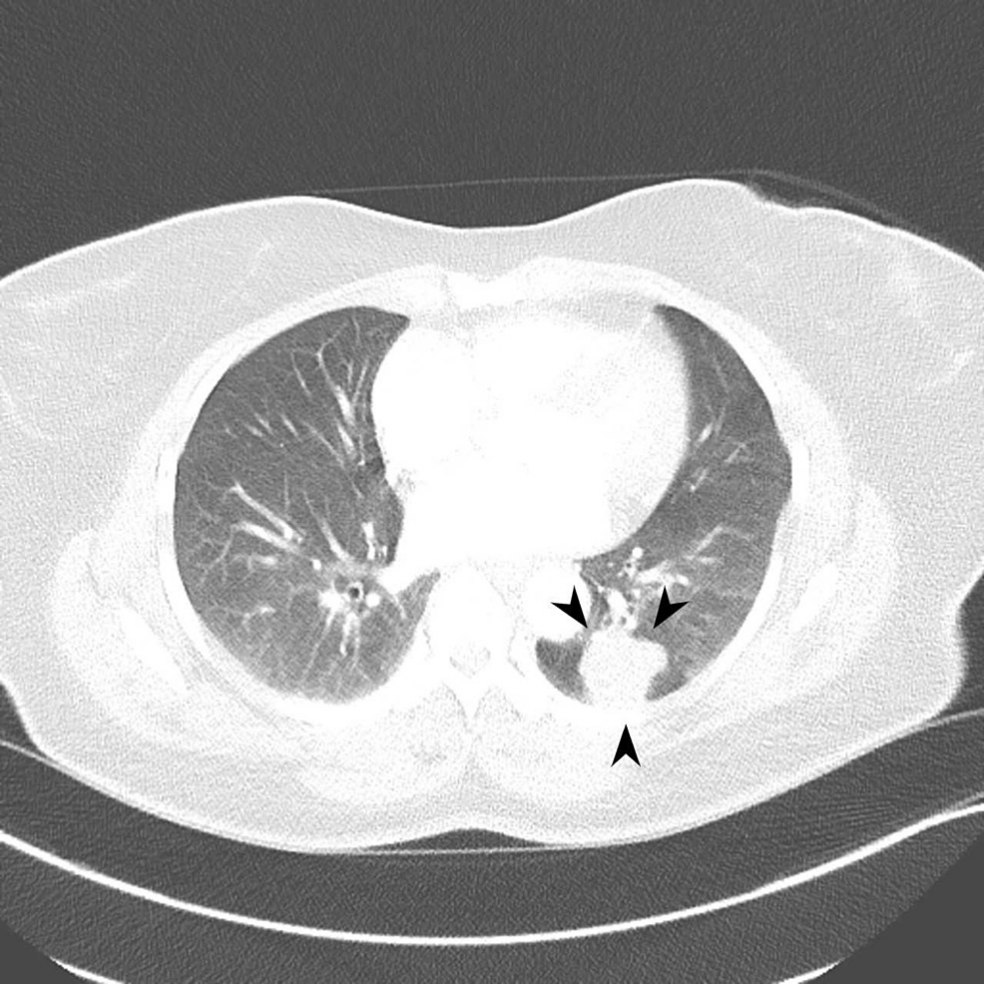
Heterogeneously enhancing 2.8 x 2.5 cm solid mass in the left lower lobe with tiny surrounding satellite nodules.

**Figure 3:**
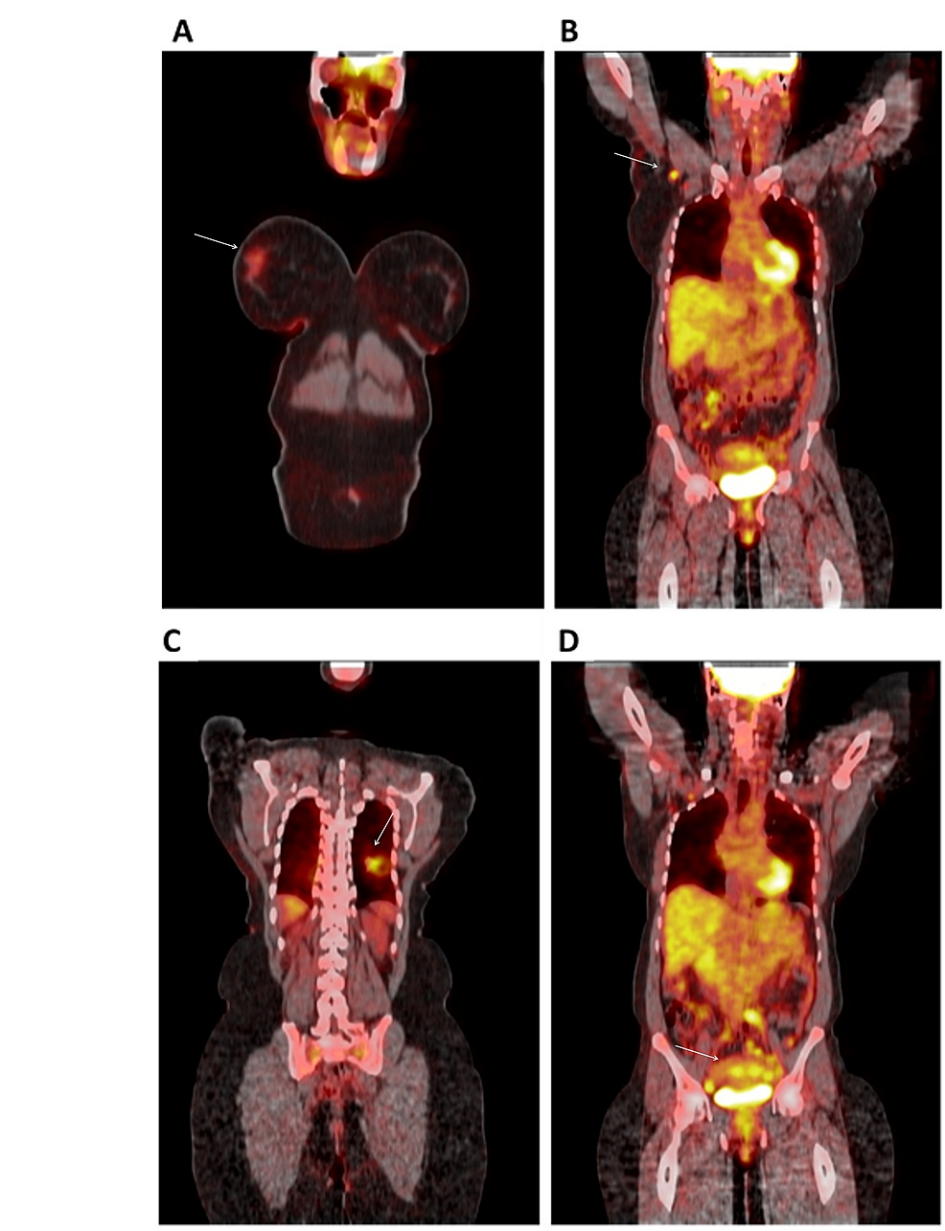
Whole-body PET scan. Panel A: FDG avidity in the right breast. Panel B: FDG avidity in the right axillary lymph nodes. Panel C: Significant enhancement in the left lower lobe mass with a SUVmax of 3.1. Panel D: FDG avidity in the uterus. PET: Positron emission tomography; FDG: 18F-fluorodeoxyglucose.

A CT scan of the chest revealed a heterogeneously enhancing 2.8 x 2.5 cm solid mass in the left lower lobe and several satellite nodules (Figure 2). The positron emission tomography (PET) scan showed significant enhancement in the mass with a SUVmax of 3.1 along with significant 18F-fluorodeoxyglucose (FDG) avidity in the right breast and axillary lymph nodes, and nonspecific avidity in the uterus (Figure 3). These findings suggested a possible primary neoplasm from the genitourinary tract, breast, or lymphoma.

A diagnostic mammogram and screening breast MRI were negative for breast malignancy. Fine needle aspiration of the axillary lymph node revealed nonspecific follicular hyperplasia and polyclonal reactive plasmacytosis. Pelvic and transvaginal ultrasounds, uterine dilation and curettage with pathologic examinations, and repeat evaluation by two separate gynecologic oncologists did not reveal any evidence of primary malignancy in the reproductive organs.

**Figure 4:**
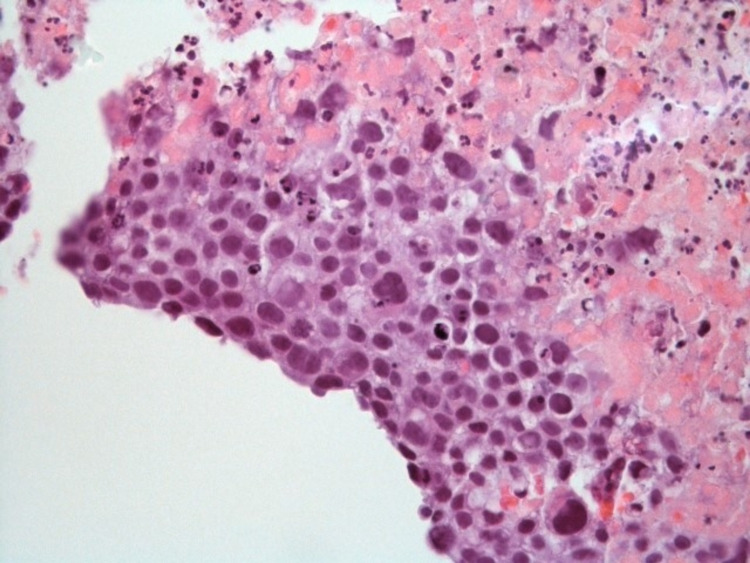
Hematoxylin and eosin staining of left lower lung tumor at 40x magnification revealing tissue that is mostly necrotic and composed of large pleomorphic epithelioid cells; some with mucin vacuoles.

**Figure 5:**
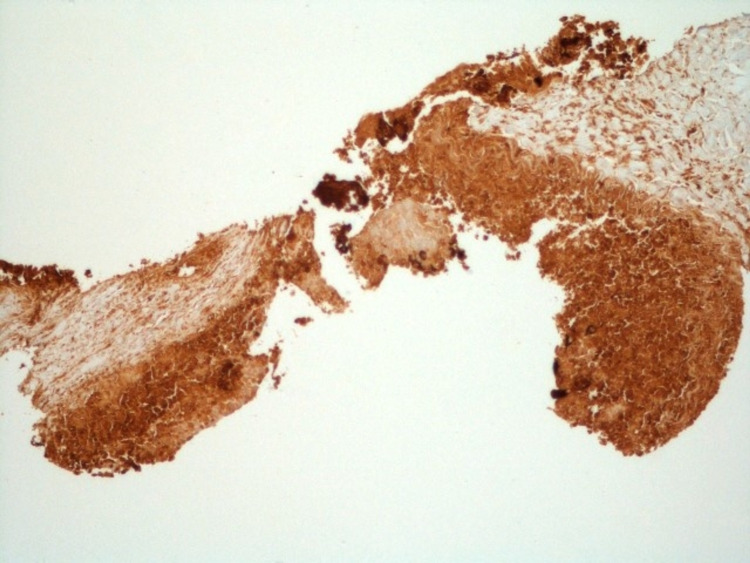
Positive Immunohistochemical staining of the tumor with β-HCG shown at 10x magnification. β-HCG: Beta-human chorionic gonadotropin.

**Figure 6:**
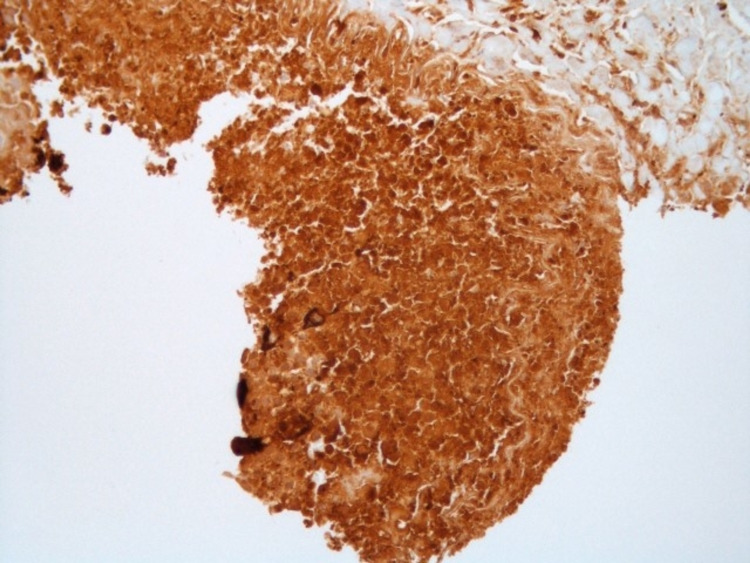
Positive immunohistochemical staining of the tumor with β-HCG shown at 20x magnification. β-HCG: Beta-human chorionic gonadotropin.

CT-guided core biopsy of the lung mass revealed a necrotic tumor composed of large pleomorphic epithelioid cells (Figure 4). Immunohistochemical staining was initially nonspecific with positive staining for GATA-3 and focally for CDX2. Staining was negative for markers of small cell and non-small cell carcinomas of the lung and metastatic breast cancer. In light of the patient’s elevated serum β-HCG, which by now had trended up to 24,860 mIU/ml, the specimen was re-evaluated with immunostaining for markers of germ cell neoplasms. It was found positive for Sal 4, pan-cytokeratin, cytokeratin 8/18, β-HCG, and rare positive staining with Glypican 1 (Figures 5-6).

**Figure 7:**
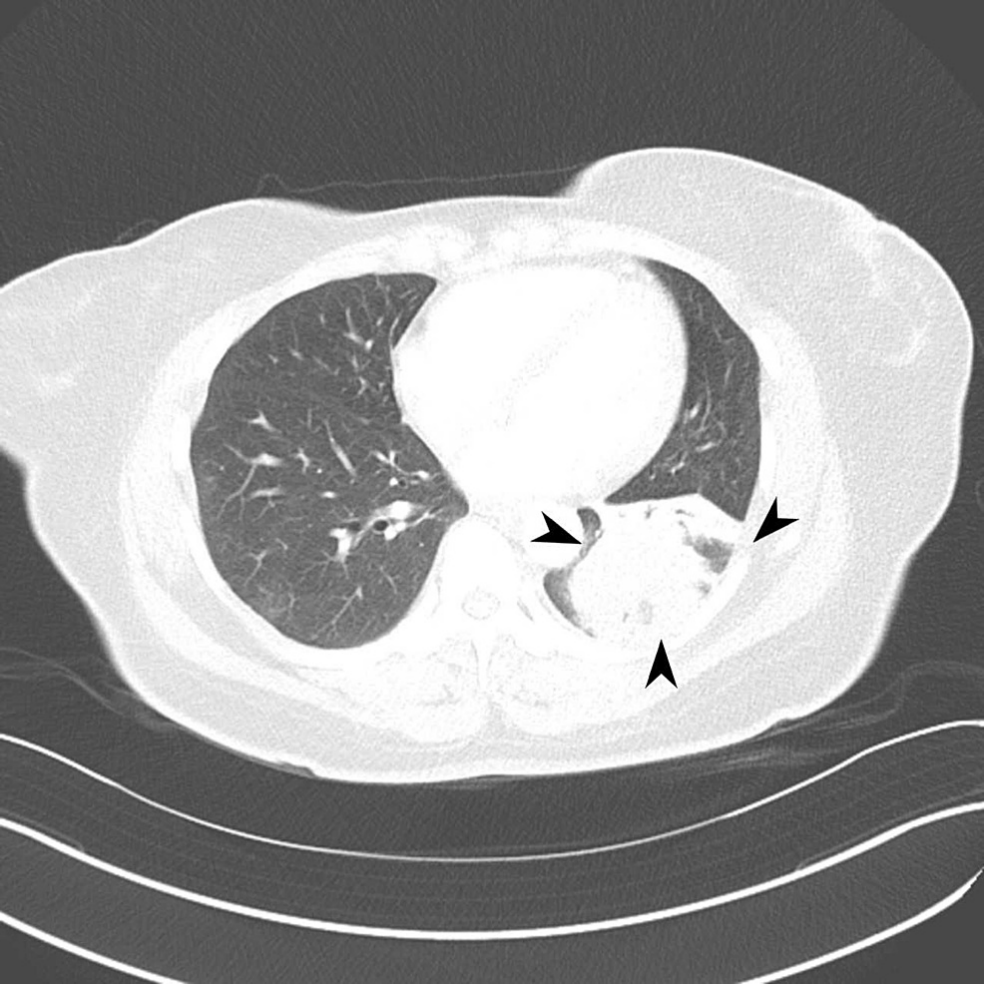
Pre-induction CT imaging of left lower lobe tumor revealing mass enlargement compared to size on presentation.

The pathologic diagnosis was made for a non-dysgerminoma, malignant GCT with choriocarcinoma component, and the patient was diagnosed with a T3N0M0 PPC. Interim chest CT scan prior to chemotherapy showed an increase in the size of the tumor to 4.8 x 3.6 cm (Figure 7). After discussions with the multi-disciplinary tumor board, the patient was started on neoadjuvant vincristine, ifosfamide, and cisplatin (VIP) chemotherapy (Table 1).

**Table 1:** Cisplatin, etoposide, and ifosfamide neoadjuvant chemotherapeutic regimen. VIP: Vincristine, ifosfamide, and cisplatin.

Therapeutic agent	Length of treatment	Dosage
VIP cycle	21 days (cycle length)	4 cycles
Cisplatin	Days 1-5	20 mg/m^2^
Etoposide	Days 1-5	75 mg/m^2^
Ifosfamide	Days 1-5	1200 mg/m^2^
Mesna	Days 1-5	1200 mg/m^2^ on day 1, 120 mg/m2 on days 2-5

**Figure 8:**
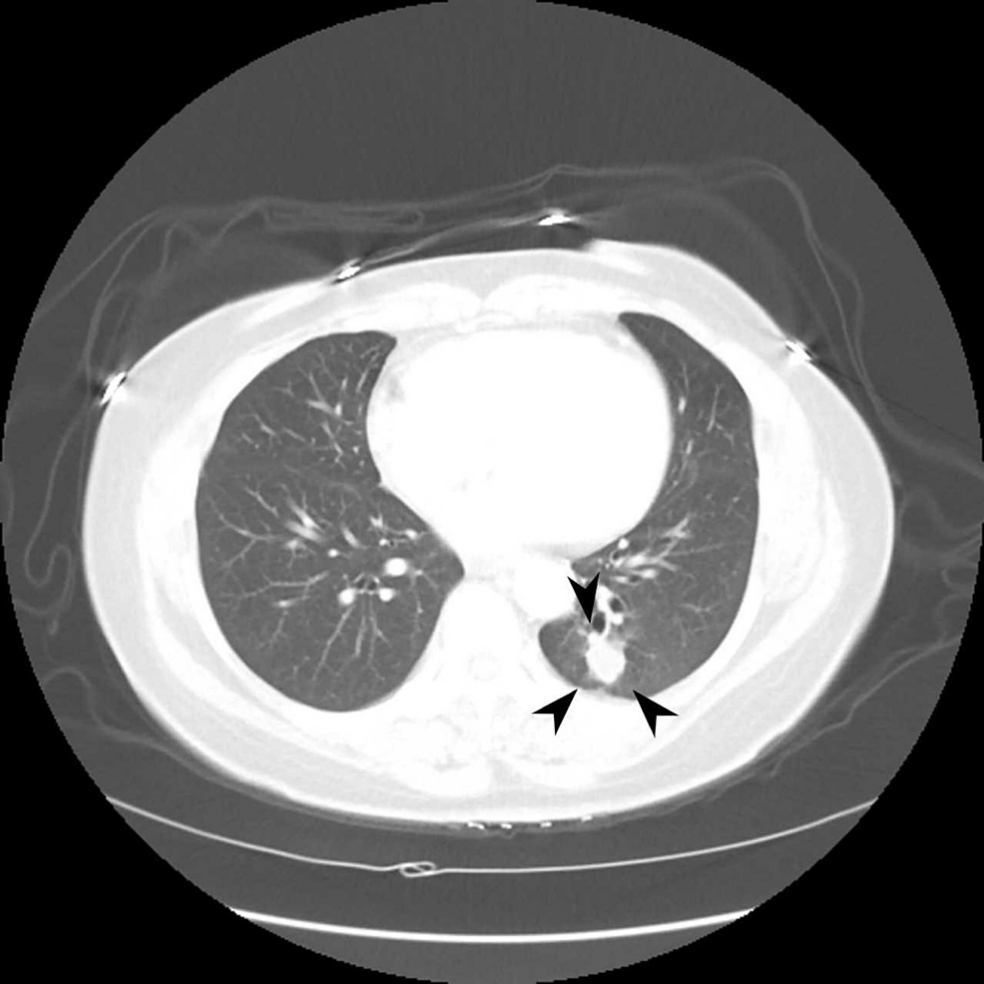
Post-induction chemotherapy CT imaging of left lower lobe tumor revealing shrunken mass.

β-HCG level prior to systemic chemotherapy was 98,138 mIU/ml, and after the completion of four cycles of VIP, it reduced to normal levels (4 mIU/ml). A CT chest with IV contrast after completion of the fourth cycle showed a significant decrease in the size of the tumor to 2.1 x 1.6 cm (Figure 8).

Six weeks after completing induction chemotherapy, the patient underwent video-assisted left thoracoscopic surgery (VATS) with lower lobectomy and mediastinal lymph node dissection. Surgical margins and ten lymph node stations were negative for tumor and nodal metastases, respectively. The patient had a complete pathologic response with no residual viable tumor cells. Postoperative recovery was uneventful, and the patient was in remission at four-month postoperative follow-up with undetectable β-HCG.

Despite these favorable early results, the patient later presented with relapsing metastatic disease. Approximately nine months after completion of treatment, she presented with new pulmonary nodules and brain metastases. Pathologic examination of the brain lesion was consistent with metastatic choriocarcinoma. Her β-HCG level had also increased to 160 mIU/ml. Despite treatment with adjuvant radiation, her prognosis remains poor.

## Discussion

PPC is a rare form of extragonadal malignant GCT that requires the following diagnostic criteria: (1) exclusion of primary lesions in gonads, (2) normalization of β-HCG levels after treatment, and (3) confirmation by pathology [4]. To distinguish from giant cell carcinoma, PPC typically stains negative for TTF-1. Of note, our patient presented postmenopausal, with no recent pregnancy or abortions, indolent symptoms for at least six months, an elevated β-HCG for two years prior to symptoms, and an enlarging mass in the left lower lobe. Most cases of choriocarcinoma occur after a gestational event. Furthermore, non-gestational choriocarcinoma is usually seen in children and young adults [5].

Diagnosing PPC is imperative as PPC is known to carry a poor prognosis due to early hematogenous dissemination. The tumor markers α-fetoprotein and β-HCG are essential in managing GCTs. Due to their specificity and sensitivity, they are useful for the initial diagnosis and for the monitoring of treatment along with lactate dehydrogenase [6-7]. Our case suggests that an idiopathically elevated β-HCG in a patient with a lung mass or nodule with a high probability of malignancy should prompt the addition of stains for GCTs to the more commonly used stains during pathologic examination.

There is no standardized protocol for the treatment of PPC, and there are currently no guidelines as to the proper combination of treatment modalities such as chemotherapy, radiation, and surgery. However, case reports and systematic reviews have focused on achieving remission by combining surgery and chemotherapy regimens adopted from the treatment of high-risk GCTs. Bleomycin, methotrexate and cisplatin (BEP) and etoposide, methotrexate, actinomycin D, cyclophosphamide and vincristine (EMA-CO) have been tried with some success. Our patient did not undergo chemotherapy treatment with EMA-CO or BEP. VIP treatments are another therapeutic option for patients with advanced, gonadal, or extragonadal poor-risk GCTs and are preferred over BEP for mediastinal nonseminomatous GCTs. These patients undergo resection of residual disease with prolonged exposure to oxygen during surgery which can provoke bleomycin-related pneumonitis. Treatment with BEP was considered, but VIP was preferred to avoid this complication that can lead to acute respiratory distress syndrome (ARDS) and higher rates of prolonged postoperative ventilator dependence [8-12].

## Conclusions

PPC is a rare lung tumor that should be considered in the appropriate clinical context. Serum β-HCG levels and tissue immunohistochemical stains are essential for diagnosis. To the best of our knowledge, this is the first reported case of PPC that was treated with VIP induction chemotherapy. Longer follow-up and information from other cases will be needed for evaluating outcomes in patients with PPC treated with VIP. Careful consideration of the possible adverse effects of agents such as bleomycin-related pneumonitis is warranted when choosing a chemotherapeutic regimen. Further investigation into proper treatment regimens, as well as factors influencing survival, is needed in order to improve clinical outcomes in patients with PPC.
